# The Effect of Encapsulation on Crack-Based Wrinkled Thin Film Soft Strain Sensors

**DOI:** 10.3390/ma14020364

**Published:** 2021-01-13

**Authors:** Thao Nguyen, Michael Chu, Robin Tu, Michelle Khine

**Affiliations:** 1 Department of Chemical and Biomolecular Engineering, University of California, Irvine, CA 92697, USA; thaon8@uci.edu; 2 Department of Biomedical Engineering, University of California, Irvine, CA 92697, USA; mchu8@uci.edu; 3 Department of Statistics, University of Illinois, Urbana-Champaign, Champaign, IL 61820, USA; robintu2@illinois.edu

**Keywords:** wearable technology, strain sensor, soft wearable sensors, polymer encapsulation

## Abstract

Practical wearable applications of soft strain sensors require sensors capable of not only detecting subtle physiological signals, but also of withstanding large scale deformation from body movement. Encapsulation is one technique to protect sensors from both environmental and mechanical stressors. We introduced an encapsulation layer to crack-based wrinkled metallic thin film soft strain sensors as an avenue to improve sensor stretchability, linear response, and robustness. We demonstrate that encapsulated sensors have increased mechanical robustness and stability, displaying a significantly larger linear dynamic range (~50%) and increased stretchability (260% elongation). Furthermore, we discovered that these sensors have post-fracture signal recovery. They maintained conductivity to the 50% strain with stable signal and demonstrated increased sensitivity. We studied the crack formation behind this phenomenon and found encapsulation to lead to higher crack density as the source for greater stretchability. As crack formation plays an important role in subsequent electrical resistance, understanding the crack evolution in our sensors will help us better address the trade-off between high stretchability and high sensitivity.

## 1. Introduction

Soft, stretchable strain sensors have attracted much interest for human application as they can retain functionality while enduring large deformations and remain conformal to the body. They have been applied in rehabilitation [1], motion detection [2,3], wearable health monitoring [4,5], and facial expression detection [6]. As most conductive materials tend to be rigid, a common approach to increase stretchability is to incorporate wrinkled, serpentine, cracked, or mesh structures for strain relief [7] and support the functional layer with an elastomeric substrate. These sensors, however, can be susceptible to physical damage during use and must withstand mechanical handling. One method to protect the more delicate functional layer is to introduce an encapsulation layer to aid in mechanical robustness for prolonged application and handling.

Specifically, piezoresistive sensors produce a change in electrical resistance when stretched or compressed. Structural changes can come from geometric considerations such as disconnection mechanisms, crack propagation across a thin film, or the electron tunneling effect through thin polymer layers [8]. In particular, crack-based strain sensors are of increasing interest for the high signal sensitivity that can be achieved as more subtle motions such as respiration or pulse would require this for accurate detection. Kang et al. initially reported on an ultrasensitive mechanical crack-base sensor inspired by the crack-like slits in spiders for a platinum (Pt) thin film on polyurethane acrylate that demonstrated a sensitivity of 2000 for the 2% range [9]. Others have also leverage nanometallic thin films for ultrahigh sensitivity at low strain ranges (<5% strain) [10,11,12]. This working stretchability range, however, is unsuitable for physiological relevant strain ranges such as human motion (>50% strain [13,14]). Stretchable strain sensors often encounter a trade-off between high sensitivity and high stretchability. The former favors a functional element that undergoes large structural changes under small strains, whereas the latter requires the conductor to maintain a conductive pathway with large deformation [15].

Researchers have explored strategies to expand this narrow sensing range while still leveraging the high sensitivity of a crack-based mechanism. Sensitivity is defined by gauge factor, GF = (ΔR/R_0_)/ε, where ∆R is the change in resistance, R_0_ is the initial nominal resistance, and ε is the applied strain. Amjadi et al. reported a graphite thin film sensor that achieved a gauge factor of 522.6 at 50% strain by exposing the elastomeric substrate to oxygen plasma prior to depositing the thin film, generating parallel microgrooves within the film [16]. This technique, however, also significantly stiffens the polymer substrate and limits its stretchability at 50% strain; the sensor was reported as no longer conductive past this strain point. Jeon et al. presented a Pt-based strain sensor with high crack density for the measurement of whole-body human motions (>100% strain) [16,17], reporting an initial gauge factor of 30 at 50% strain for a 10 nm thickness. They extended that working range to 150% strain, but required the deposition of more platinum [14]. Previous work done in this lab has achieved a gauge factor of 42 with a maximum dynamic range of 182% [4]. Others offer potential improvements on electromechanical reversibility, reproducibility, and durability with additional encapsulation at the expense of sensitivity [5,17,18,19]. Encapsulation will be necessary in use cases where additional durability is required (e.g., during exercise).

Here, we introduced an encapsulation layer to our wrinkled metallic thin film soft strain sensor and characterized its impact on the electromechanical performance. A study of relevant clinical application in respiration using encapsulated sensors has been demonstrated in a separate publication [5]. We show that introducing an encapsulation layer not only protects from physical damage and environmental stressors, but also increases sensor mechanical robustness and stability. With encapsulation, the sensor displayed a significantly larger linear dynamic range (~50%) and increased stretchability (260% elongation). Moreover, the encapsulated sensors also had recoverable electrical signal with reliable functionality post-fracture. After they had been stretched to electrical failure, they could maintain conductivity to 50% strain with stable signal and displayed increased gauge factor. Often, electrical recovery is most commonly discussed as a static state where, upon unloading the applied strain, the sensor is allowed to return to its original length [20,21]. We investigated the impact of the encapsulation layer on the crack mechanism and studied the contribution of crack formation to the electromechanical performance of our soft strain sensors. To the best of our knowledge, this is the first paper to investigate sensor encapsulation along with the mechanics of crack evolution and post-fracture recovery. Although the additional polymer layer impacts signal sensitivity and recovery time, reliability and durability are meaningful properties in active use case; certain applications (e.g., those in an aqueous environments) are impossible with an exposed conductive element.

## 2. Materials and Methods

Unencapsulated sensors were fabricated based off a previously reported technique for patterning metallic thin films onto shape memory polymers [4]. To pattern the sensor design, the desired geometry was created using a computer aided design software (AutoCAD, 22). This design was then laser etched into a one-sided adhesive tape mask (Grafix Arts, Frisket Film, Maple Heights, OH, USA). This mask was then applied onto a pre-stressed shape memory polymer (SMP), polystyrene (PS), substrate. We deposited a 5 nm Pt thin film onto the masked substrate with a timed deposition (207 s) in a magnetron sputter coater (Quorum Technologies, Q150R, Laughton, East Sussex, UK). Next, 5 nm of gold (Au) was then deposited, also using a timed run (102 s). Gold was used as an adhesion layer to chemically bind with a silane treatment to promote molecular adhesion to the subsequent elastomer layer later [22]. The tape mask was then removed, leaving the sensor design on the PS. The metal-deposited SMP was then heated past its glass transition temperature (100 °C) in a convection oven set to 140 °C for 13 min, causing it to shrink roughly 67% in area [23]. The stiffness mismatch between the metallic thin film and the substrate causes the film to buckle and form hierarchical wrinkled structures [24]. The sample is then immersed in a 5 mM (3-mercaptopropyl) trimethoxysilane (95% MPTMS) ethanol solution for 1 h at room temperature to functionalize the Au surface. After silane treatment, a silicone elastomer (Smooth-on, Ecoflex 0030, Macungie, PA, USA), was immediately spin coated onto the sample at 150 rpm for 35 s before thermal curing for 2 h at 80 °C. This resulted in a substrate thickness of 700–800 µm. The sensor was then lifted off the PS via an acetone bath followed by a toluene wash, immediately rinsed with acetone, and allowed to air dry.

**Figure 1:**
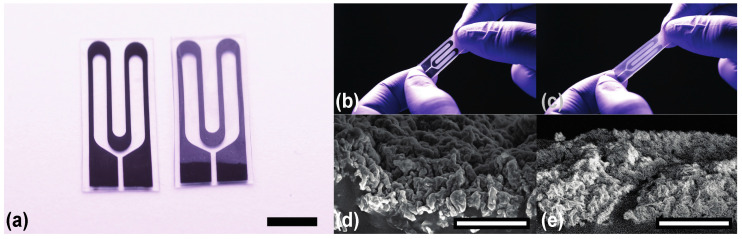
(**a**) Fabricated unencapsulated and encapsulated sensors prior to characterization. Scale bar is 5 mm. (**b**) Unencapsulated sensor unstrained (0% strain). (**c**) Unencapsulated sensor strained roughly to 50% strain. (**d**) Scanning electron microscope (SEM) image of the wrinkle features unstrained (0% strain) (taken with secondary electrons) and (**e**) strained (50% strain) (taken with backscattered electrons). Scale bar in SEM images is 10 μm.

Encapsulated sensors, once dried, were spin coated with the same silicone elastomer, Ecoflex 0030, at 1000 rpm for 35 s, resulting in an encapsulated thickness of ~30 µm, and left to cure for 2 h at 80 °C. A schematic of the fabrication process is provided in the Supplementary Figure S1. The final form of each sensor type is shown in Figure 1a. Demonstration of the wrinkled thin film unstrained and strained are shown in Figure 1b,d and 1c,e, respectively. Exposure to organic solvents during the lift off process caused the elastomeric substrate to visibly swell while wet, but the overall morphology was still preserved and can be seen post-transfer in the cross-sectional scanning electron microscopy (SEM) image in Figure 1d. Figure 1d was taken with secondary electrons to better depict the topography differences in the wrinkled features whereas Figure 1e was taken with backscattered electrons to better visualize the presence of cracks in the wrinkled film under applied strain as this detection source is preferred for observing chemical composition differences (i.e., polymer vs. metal).

## 3. Results and Discussion

### 3.1. Electromechanical Characterization

**Figure 2:**
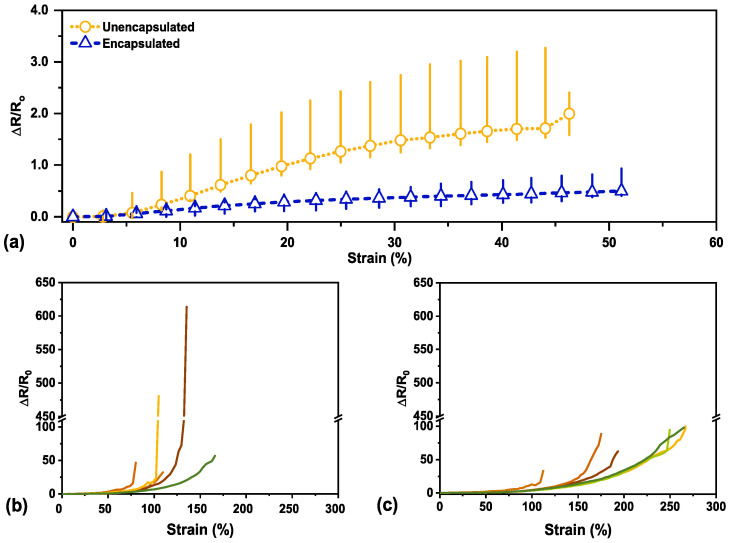
(**a**) Electromechanical response for unencapsulated and encapsulated sensors, respectively, tracks the normalized change in resistance (ΔR/R_0_) with applied strain. The marker indicates the median value with the bar depicting the range from minimum to maximum across N = 6. (**b**) Strain-to-failure behavior for each unencapsulated sensor, N = 6, and for each encapsulated sensor (**c**), N = 6, to study stretchability.

The electrical response to tensile strain is depicted for unencapsulated and encapsulated sensors in Figure 2a. Each sensor type was tested for a physiologically relevant tensile strain range [13]. The minor variation in the depicted strain range for each sensor seen in Figure 2a comes from the distance measurement error of the testing apparatus. The stretchability and dynamic range of both sensor types were also observed with strain-to-failure testing (see Figure 2b,c).

Overall, the unencapsulated sensors indicate a higher sensitivity to tensile strain with a median GF of 4.3 at 45% strain (with a range of 3.4 to 5.2), whereas in comparison, the encapsulated sensors had a median GF of 1.0 (ranging from 0.66 to 1.7) at 45% strain. Although the samples will shrink roughly 67% in area, the shrinking is not entirely uniform and can cause some variation in the final sample size. Moreover, the samples will shrink further with organic solvent exposure (~10–15% more). Variety in sample size along with minor mounting differences into the testing apparatus can contribute to the spread in sensitivity data observed in Figure 2a. The change in signal for each sensor was normalized in order to be comparable. The sensitivity characterization and data for all unencapsulated and encapsulated sensors can be found in Supplementary Figures S2–S4.

Previously, we hypothesized that our wrinkled thin film resistance change under strain was primarily caused by the adjacent wrinkle structures separating as the sample elongates [25,26,27]. At moderate to high strain, fractures begin to form, causing resistance to increase as the fractures elongate close to maximum strain [4]. Although the gauge factor was lower for the encapsulated sensors, the average working range for our sensors increased with encapsulation, as shown in the strain-to-failure characterization in Figure 2c. The variation in behavior for both sensor types at high strain (>75%) is likely due to natural variation in fracture nucleation and propagation pathways with applied load. Higher sensitivity, or an increased change in resistance, indicates that cracks have appeared within the film whereas with small resistance change, there was insignificant mechanical damage within the film [28,29,30,31]. We hypothesize that the decrease in sensitivity is due to the stress being further delocalized into the encapsulated polymer layer, preventing concentrated localized mechanical stress in the thin film, which will delay the onset of fractures forming in the wrinkled thin film and inhibit large crack growth once cracks have formed at higher strain. This theory is later visually investigated in Section 3.5 (Crack Evolution and Sensor Mechanism). The presence of an encapsulation layer would also provide additional mechanical support for the metallic thin film as it would physically prevent the film from fully delaminating from the substrate.

### 3.2. Signal Latency

**Figure 3:**
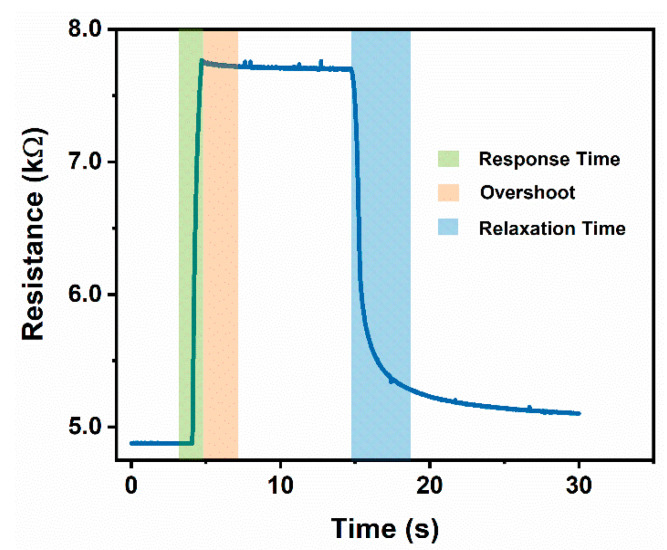
Representative schematic of segments used to determine response time, overshoot, and relaxation time of each sensor with segments based off a 90% time constant. All polymer-based strain sensors have a response delay due to the viscoelastic nature of the polymer.

Signal latency metrics such as response time, signal overshoot behavior, and relaxation time of our sensors are important parameters for practical use as wearable sensors. A schematic to help visualize the signal behavior for these metrics can be found in Figure 3. It is important to note that all polymer-based strain sensors have a response delay due to the viscoelastic nature of the polymer; an appropriate response time value for these sensors has been established at a 90% time constant [4,17,20]. We reported an average response time of 29 ± 5 ms for the unencapsulated sensors and 34 ± 5 ms for the encapsulated sensors, indicating that encapsulation did not cause a significant (*p*-value 0.1139) latency delay on our sensor response. Sheridan and Ferrell reported the maximum latency to be classified as “no delay” by human subject tests as 45 ms [32]. Relaxation time upon releasing an applied load is often dominated by the stress relaxation of the polymer, making it prone to a recovery delay. A 90% time constant is also commonly reported for relaxation time. Our sensor relaxation time also suffered from the viscoelastic effects of the polymer being exposed to organic solvents for both the unencapsulated and the encapsulated sensors. The additional relaxation time in the encapsulated sensor can be attributed to the added relaxation time of the cross-linked encapsulation layer and the wrinkled thin film (on order of seconds) [33,34,35]. Overshoot behavior can also be quantified for polymer-based sensors where a set strain is applied and held constant over time. The average reported values for each sensor type can be found in Table 1.

### 3.3. Post-Fracture Characterization

These strain sensors were still functional past 50% strain, even after they had been stretched to electrical failure, defined as post-fracture, and even exhibited increased sensitivity. By straining the sensors to electrical failure first, it is implied that we introduced a catastrophic crack within our functional thin film. Similar to how we can use preconditioning to introduce microcracks, straining the sensors to electrical disconnection is a more aggressive form of increasing the resistance in our film. From the literature, increasing the electrical resistance of polymer supported metal films during tensile testing is the result of two main contributions: geometrical and structural [28,36,37,38]. Geometric considerations are from increasing the physical distance between contact points as the sample is elongated as well as the simultaneous compression of the sample in the transverse direction due to the Poisson’s ratio. Structural contributions include point defect density, grain boundary density, cracking, necking (local thinning), dislocation pileups, or intrusions [37]. By straining the sensors to the electrical failure point first, we can aggressively increase our signal sensitivity. Although we introduced a combination of physical defects in our thin film, the hierarchical wrinkle features enable for a conductive pathway to remain at strain ranges below the failure strain point, even as these defects broaden and elongate with applied strain.

### 3.4. Durability

Electrical signal degradation has been used as the failure criterion for the study of a material’s lifetime and reliability [28]. We studied the cycling behavior of the pre-fractured sensors, observing the tensile cycling to 50% strain at 4 mm/s for 5000 cycles. The samples were initially preconditioned to 100% strain for 100 cycles (not shown) prior to continuous cycling to 50% strain for 5000 cycles to reflect the preconditioning in our sensors under use. We preconditioned our sensors to deliberately induce cracks within the thin film by straining it at a higher strain point than the intended working strain range to distribute microcracks across the film without causing cracks to fully propagate. Introducing these microcracks prior to experimental application allows the film to deform elastically under larger strains rather than inducing plastic strain (with the initial onset of cracks) under use [39]. Durability behavior without prior preconditioning can be found in Supplementary Figure S5.

**Figure 4:**
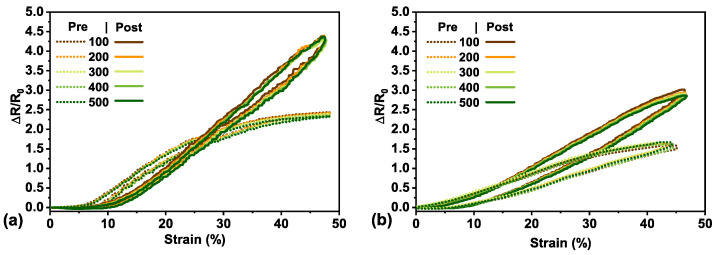
Cycling behavior of an unencapsulated (**a**) and encapsulated sensor (**b**), respectively, with pre-(dotted lines) and post-fracture (solid lines) represented every 100th cycle.

The sensor behavior remained stable throughout the duration of the test, displaying very little signal deviation across cycles. Figure 4 displays representative pre-fracture cycling behavior (dotted lines) for the unencapsulated (Figure 4a) and encapsulated (Figure 4b) strain sensor, respectively. Every 100th cycle is shown with the first cycle not depicted as it does not accurately represent the sensor performance. We attributed this to the Mullins effect where the electromechanical signal was dominated by the mechanical behavior of the elastomeric substrate. The Mullins effect is a phenomenon observed in rubber-like materials (elastomers) and describes cyclical stress softening as a result of the evolution of hard and soft domain microstructures within the material, irreversible damage within the material, or a combination of both [40]. The most pronounced softening occurs between the first and second cycle; after a few cycles (5–10 is the most commonly reported in the literature), the material response of the subsequent cycles concurs. Any additional softening after is from the effect of fatigue [41]. The full cycling data for both unencapsulated and encapsulated sensors can be found in Supplementary Figures S6 and S7.

In comparison, to observe the stability of the sensor post-fracture, each sensor type was cycled once again to 50% strain for 500 cycles. As with the pre-fractured cycling, the initial cycle was always observed to be different from the subsequent cycles. Again, we attributed this to the Mullins effect where rubber-like materials have an observed cyclical signal softening in response to deformation [41]. The observed cycles at every 100th cycle for post-fractured sensors can be seen in Figure 4 (solid lines) for the unencapsulated (Figure 4a) and encapsulated (Figure 4b) sensors. The full post-fracture cycling data can be found in Supplementary Figures S8 and S9. There was little to no change in resistance observed for the <10% strain in Figure 4a and <5% strain in Figure 4b, which was most likely due to mounting the sensor slightly less than taut initially. However, response degradation and softening can also be attributed to fatigue, along with observed plastic deformation of the elastomeric substrate [4,17,42,43].

Furthermore, the average post-fracture gauge factor at 50% strain could also be quantified from this cycling data and compared to that of the pre-fractured sensors. The unencapsulated sensitivity displayed a 2.4× increase (GF from 4.3 to 10.5), whereas the encapsulated showed a 5.4× increase (GF from 1 to 5.4). This can likely be attributed to the structural changes (point defects, cracks, necking, dislocation pileups, and intrusions) introduced by straining to a maximal electrical point along with the additional repeated loading and unloading cycles. The hierarchical wrinkles within the thin film would also contribute to the random dislocation pileups and intrusions with loading and unloading, having an effect on the distribution of contact points. Although the unencapsulated sensors still had higher sensitivity than the encapsulated sensors, they are more subjected to physical damage with handling and are far less reliably conductive post-fracture. All the encapsulated sensors tested remained reproducibly conductive post-fracture whereas only a portion (two thirds) of the unencapsulated sensors were still conductive for the full cycling to 50% strain. It is most likely that the encapsulation layer physically protects the thin film from further damage due to environmental factors or handling. We theorize that the encapsulation layer physically inhibits further large crack widening within this working range (as we are operating well below the established failure strain point) as the additional polymer layer bears some of the load with applied strain, preventing concentrated stress in the thin film being reached as readily. This type of crack formation is more evenly distributed throughout the thin film of the encapsulated sensors. This hypothesis was confirmed by visualizing the polymer supported thin film with bright field optical microscopy and is discussed in the next section.

### 3.5. Crack Evolution and Sensing Mechanism

#### 3.5.1. Crack Evolution

**Figure 5:**
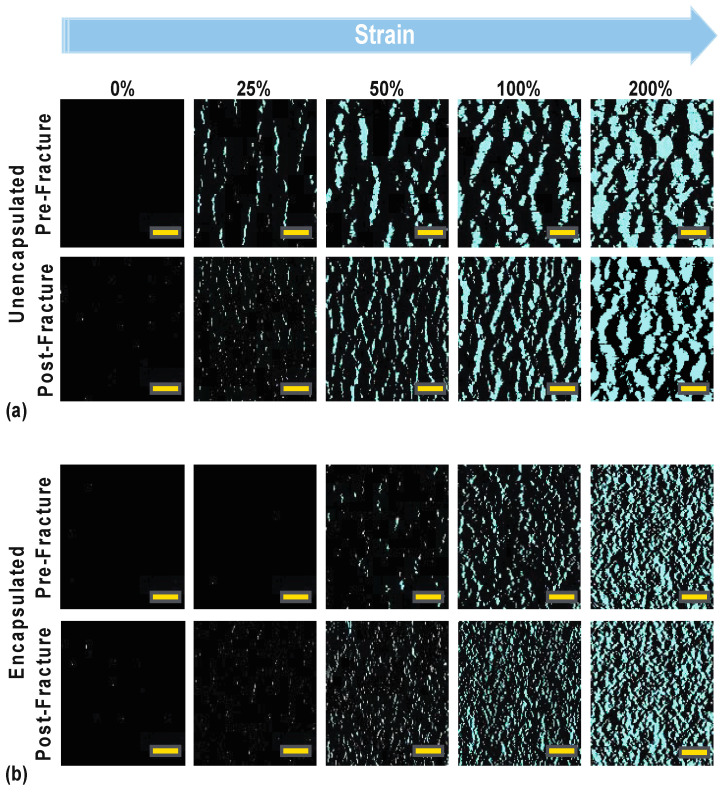
Bright field images of the crack evolution pre-and post-fracture for an unencapsulated (**a**) and encapsulated (**b**) sensor, respectively. Scale bar is 100 μm for each panel. Cracks have been pseudo-colored for better visualization purposes of this figure. All image analysis was done on non-colored, unaltered images.

Optical images were taken at set strain points to observe the crack evolution within our wrinkled thin films, as displayed in Figure 5. The cracks in these images were pseudo-colored for better visualization in the figure only. All image analysis was done on uncolored, unaltered bright field images. The crack distribution of the unencapsulated thin film under applied strain supports the previous hypothesis where the fractures begin to form at moderate to high strain and further elongate once we approach maximum strain. The unencapsulated film displayed fewer but larger cracks, as seen in Figure 5a, allowing the film to tolerate a moderate level of strain, but those cracks continued to grow and widen with increasing strain until one crack eventually propagated through the thin film to cause electrical disconnection. In comparison, we theorized that the addition of the encapsulation layer would change crack distribution through the wrinkled thin film and create more crack nucleation points to form. These small cracks eventually coalescence into larger ones with increasing strain but delay the onset of a catastrophic crack. The encapsulated film displays many smaller cracks due to strain delocalization across the entirety of the film compared to the unencapsulated film at the same equivalent strain points. Again, this delocalization helps prevent the propagation of a catastrophic crack across the film as most of the large elastomeric strain would be induced in the polymer substrate and encapsulation layer. This theory of crack evolution was confirmed in our investigation with the images in Figure 5b. Within a low-strain region (>25%), very few cracks were seen in the encapsulated, whereas the unencapsulated film already started to form minor cracks. At 50% strain, minor cracks appeared in the encapsulated film and continued to grow with increased applied strain. More pronounced crack widening was observed in the unencapsulated film across all strain points.

Crack formation (density and geometry) plays a large role in the mechanism of changing electrical resistance and how a polymer-supported metallic thin film fails [11,36,38,44,45]. Moreover, metal film adhesion to the polymer substrate will affect its ability to elastically deform under strain. Poorly bonded films largely delaminate from the substrate and behave more similarly to free-standing films, failing by strain localizations that trigger cracking at low strain levels, whereas well-bonded films allow for the load to be transferred from the film to substrate and strain localization is slowed [46]. We have previously seen this in our wrinkled thin films without an additional adhesion layer, where the thin film would delaminate from the silicone substrate under minimal strain [4]. Larger ductility (which would change the crack formation within the film versus that of more brittle behavior) is a consequence of adequate film bonding to the substrate [38]. This larger ductility translates into higher crack density [36]. Studies of crack-based mechanisms have shown that an increased crack density leads to extended stretchability and linearity and can be considered as a measure of material strength or toughness [14,47,48,49,50,51,52]. Crack density can be indirectly confirmed by comparing the number of cracks formed within the thin film for the unencapsulated and encapsulated sensors within the same field of view.

**Figure 6:**
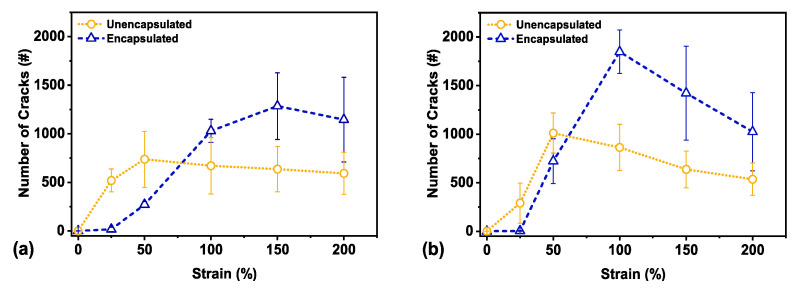
(**a**) Pre-fracture crack number for unencapsulated and encapsulated sensors, N = 3 each, and (**b**) post-fracture crack number for unencapsulated and encapsulated sensors, N = 3 each. Error bars depict standard deviation across all images. Number of cracks serve as a proxy for crack density.

As long as the observed surface area ratio of metal to overall crack area within the field of view of the taken image is the same for both unencapsulated and encapsulated samples (Supplementary Figure S10), the number of cracks formed can been used as a proxy for crack density and compared (Figure 6). Each image has a total unit area of 4.2 × 10^5^ μm^2^ or 0.42 mm^2^. The sharp increase in number of cracks at 100% strain for the encapsulated film indicates a much higher crack density in comparison to the unencapsulated film.

As previously mentioned, smaller cracks eventually coalesce into larger cracks at larger strains, as seen in the optical images in Figure 5. This most likely occurs at around 50% strain and 100% strain, respectively, as indicated by the maximal number of cracks in the unencapsulated and encapsulated sensors in Figure 6. At higher strain, crack density saturates, and the limit value is frequently used to obtain a measure of adhesion or interfacial shear strength [53]. This saturation limit is determined by the mechanical properties of the substrate along with film adhesion to the substrate [50]. Once the samples had been strained to failure and unloaded before increasing strain once again, it is likely that there is a combination of new crack nucleation within the thin film along with further coalescence of existing cracks.

To further compare the unencapsulated and encapsulated sensors, a multivariate analysis was performed on strain points of 50%, 100%, 150%, and 200% strain for each category of samples. This analysis for strain points below 50% strain was neglected as these strain points demonstrated little to no cracks to provide a substantial comparison (also seen in Figure 5). This type of comparison allows for simultaneous observation and analysis: in this case, to observe crack formation with increasing applied strain with both unencapsulated and encapsulated sensors. We used Hotelling’s T^2^ test with a directional alternative hypothesis [54] (code provided in the Supplementary Materials) and obtained a F-statistic of 13.71 with corresponding *p*-value of 0.09. While the threshold of statistical significance was set at a *p*-value of 0.05, this *p*-value still presented a 9% probability of observing these results by random chance, if the difference between the mean number of cracks of unencapsulated and encapsulated sensors was indeed zero. This *p*-value is likely the result of low power from a small sample size (N = 3 for each category), which came about from the experimental limitations.

**Figure 7:**
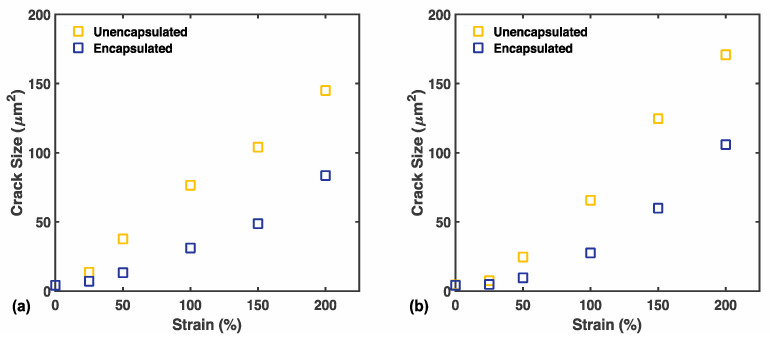
(**a**) Pre-fracture average crack area for unencapsulated and encapsulated sensors, N = 3 each. (**b**) Post-fracture average crack area for unencapsulated and encapsulated sensors, N = 3 each.

In addition to crack density, researchers may also be interested in the evolution of crack size to further tune sensor performance for a specific intended application (e.g., tracking knee rotation as opposed to respiration). Size specific characteristics of cracks are helpful in determining the appropriate stain amount to precondition sensors for an intended working range. Figure 7 shows the evolution of the average crack area of individual cracks in each case. Due to natural variation in crack nucleation and propagation, the distribution of crack size varied significantly in between images. The crack size distribution of unencapsulated and encapsulated samples are shown at each strain, for both pre-fracture and post-fracture, in Supplementary Figures S12–S15. Across all strain points, the average crack size in unencapsulated sensors was roughly double that of the encapsulated sensor.

#### 3.5.2. Sensing Mechanism

**Figure 8:**
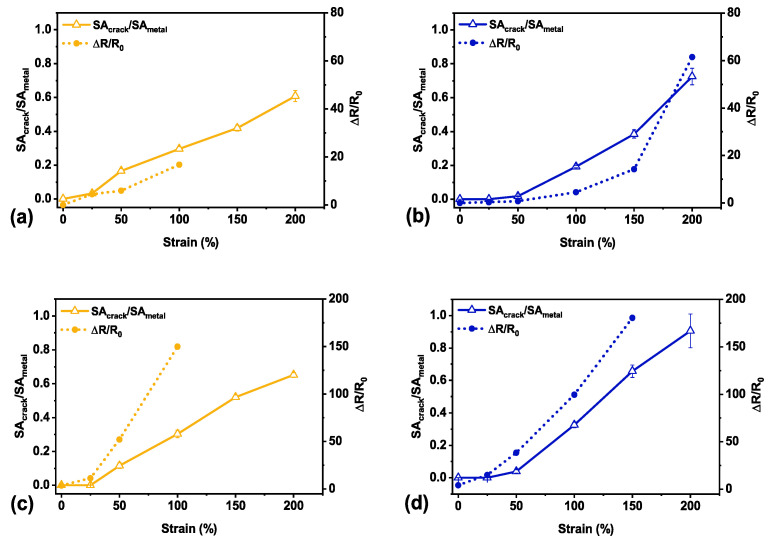
Electrical performance as related to crack evolution (SA_crack_/SA_metal_) for the best performing unencapsulated sensor, pre-fracture (**a**) and post-fracture (**c**). Electrical performance related to crack evolution for the best performing encapsulated sensor, pre-fracture (**b**) and post-fracture (**d**). Error bars on crack evolution data depict standard deviation across three separate images whereas there is only one viable measurement for electrical resistance on each sensor.

To investigate the sensing mechanism of our sensors, we simultaneously collected electrical resistance data and imaged the sensor film pre-and post-fracture at set strain points to observe how crack evolution relates to the electrical performance of the sensor. The study of crack evolution in relation to the electrical resistance is shown in Figure 8. The encapsulated sensor remained conductive to 150% strain post-fracture (Figure 8d) whereas the unencapsulated sensor only maintained an electrical signal to 100% strain both pre-fracture (Figure 8a) and post-fracture (Figure 8c) in this study. Relating the crack evolution with electrical performance allowed us to indirectly confirm the physical contribution of the encapsulated layer to the electromechanical behavior of our sensor.

Observing the performance of the pre-fracture sensors (Figure 8a,b), the ratio of crack surface area to metal thin film surface area (SA_crack_/SA_metal_) and the resulting electrical resistance remained higher for an unencapsulated sensor beyond 25% strain. As with crack evolution and failure of the polymer-supported metallic thin films, crack formation plays a large role in the mechanism of changing electrical resistance [11,36,38,44,45]. Without an encapsulation layer, straining the sensor allowed the formed cracks to continually widen with increased strain (as evident in the crack evolution imaged in Figure 5a). As the edges of the cracks separated further with strain, the resistance consequently sharply increased with applied strain [8]. It is also interesting to note that the strain point for electrical failure happened at roughly double the strain point of the peak number of cracks for the sensors studied in both cases. For the unencapsulated sensor studied, the peak number occurred at 50% strain with the last observable conductive point at 100% strain. In comparison, the encapsulated sensor showed the peak number of cracks at 100% strain and remained electrically conductive to 200% strain, pre-fracture. The number of cracks for an encapsulated sensor was also nearly double that of the unencapsulated (Figure 6). The delayed increase in crack surface area ratio coupled with a much higher number of cracks at the same strain points gave encapsulated sensors a higher crack density, and thus high adhesion and interfacial shear strength [53]. This supports similar observations in the literature where an increased crack density led to an increased failure strain point [11,14,20,47,48,49].

## 4. Conclusions

We report on the electromechanical characteristics of crack-based soft wrinkled metallic thin film sensors. The addition of an encapsulation layer provided improved mechanical robustness and stability to our sensor. We investigated the physical contribution of the encapsulation layer to the electromechanical performance: as the encapsulation layer allows for higher crack density, these sensors are able to strain further prior to electrical failure. Peak crack density is also an indication of film adhesion to the substrate along with interfacial shear strength [55]. Furthermore, these sensors are functional past electrical failure. Not only do they still have a subsequent operable stable working range, but also show increased sensitivity post-fracture as long as we remain below that fracture strain. This is attributed to the encapsulation layer delocalizing strain from the thin film and into the polymer layer, resulting in a different crack formation with increased strain and causing a divergent crack evolution from that of the unencapsulated film. The presence of an encapsulation layer allows for additional physical mechanical support and results in higher adhesion between the wrinkled thin film and polymer substrate. In doing so, we were able to leverage both the improved mechanical robustness and the crack evolution to increase our sensitivity, which would offer advantages for future use in wearable application.

Understanding how crack formation impacts sensor performance enables researchers to further tune crack-based soft strain sensors for future application. For greater utility, sensors must first be durable with real world functionality before additional tuning for specific sensor performance metrics. Ultimately, having more durable sensors enhances sensor lifetime use, allowing for long-term wear times associated with continuous monitoring and ensuring that the sensor endures repeated application cycles throughout future verification testing and validation studies.

## Figures and Tables

**Table 1:** Average reported latency values with standard deviation for unencapsulated and encapsulated sensors, respectively, with N = 6 for each category.

Sensor	Response Time (s)	Overshoot (%)	Relaxation Time (s)
Unencapsulated	0.029 ± 0.005	2 ± 2	1.1 ± 0.3
Encapsulated	0.034 ± 0.007	8 ± 7	3.7 ± 1.8

## Data Availability

The data presented in this study are available in this article and accompanying Supplemental Material. Any additional data presented in this study are available on request from the corresponding author.

## Supplementary Materials

The following are available online at https://www.mdpi.com/1996-1944/14/2/364/s1, Figure S1: Schematic of fabrication process flow, Figure S2: Representative full sensitivity curve data, Figure S3: All sensitivity curves for unencapsulated sensors, Figure S4: All sensitivity curves for encapsulated sensors, Figure S5: Full cycling data without preconditioning, Figure S6: Full pre-fracture cycling data for an unencapsulated sensor, Figure S7: Full pre-fracture cycling data for an encapsulated sensor, Figure S8: Full post-fracture cycling data for an unencapsulated sensor, Figure S9: Full post-fracture cycling data for an encapsulated sensors, Figure S10: Comparison of total surface area ratio of cracks to metal within the same field of view, Figure S11: Plot of mean number of cracks for statistical comparison, Figure S12: Distribution of crack size (crack area μm^2^) for the pre-fractured unencapsulated film across strain points, Figure S13: Distribution of crack size (crack area μm^2^) for the pre-fractured encapsulated film across strain points, Figure S14: Distribution of crack size (crack area μm^2^) for the post-fractured unencapsulated film across strain points, Figure S15: Distribution of crack size (crack area μm^2^) for the post-fractured encapsulated film across strain points. Multivariate analysis R code: Supplemental_crack_analysis_final.Rmd Details on multivariate analysis: Supplemental_crack_analysis_final.pdf
