# Supplementary material for: The Effect of Encapsulation on Crack-Based Wrinkled Thin Film Soft Strain Sensors
Source: Materials (Basel). 2021 Jan 13;14(2):364. doi: 10.3390/ma14020364 (PMC7828450; doi:10.3390/ma14020364)
Supplement: Supplementary file 1 [file materials-14-00364-s001.pdf]

materials MDPI

Supplementary Materials

The Effect of Encapsulation on Crack-Based Wrinkled Thin Film Soft Strain Sensors

Thao Nguyen [1], Michael Chu [2], Robin Tu [3] and Michelle Khine [2,*]

1 Department of Chemical and Biomolecular Engineering, University of California, Irvine, CA 92697, USA; thaon8@uci.edu
2 Department of Biomedical Engineering, University of California, Irvine, CA 92697, USA; mchu8@uci.edu
3 Department of Statistics, University of Illinois, Urbana-Champaign, Champaign, IL 61820, USA; robin-tu2@illinois.edu
* Correspondence: mkhine@uci.edu

Experimental Section

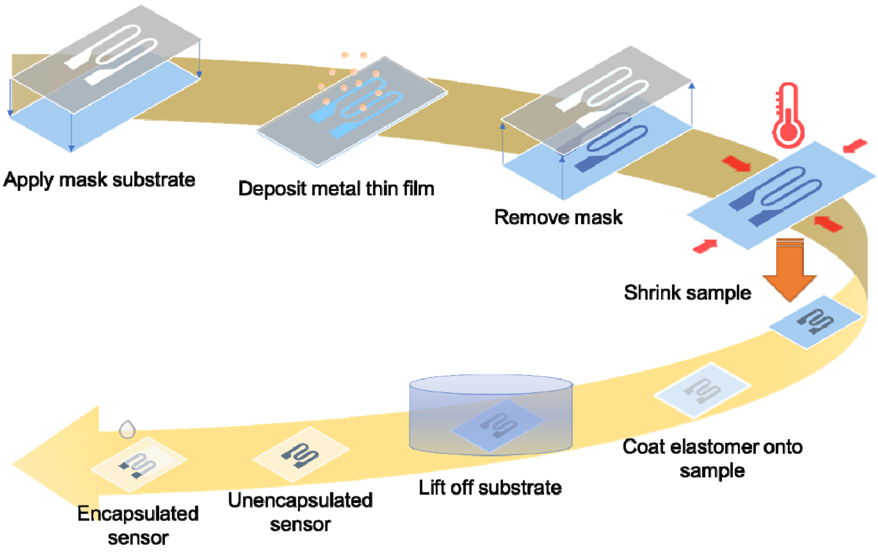

Figure S1. Process flow of sensor fabrication.

Electromechanical Characterization

The sensors are characterized using an in-house tensile testing apparatus. All sensors were initially pre-conditioned at 100% strain for 100 cycles prior to sensor characterization in order to allow for stable crack formation within the thin metal film [1]. Once conditioned, they undergo an electromechanical protocol where strain sensitivity is determined for 0% to 50% strain, pulled semi-statically with 10 seconds dwell, for 3 continuous cycles. After the strain sensitivity testing, the sensors were then subject to a response latency study. Response time, signal overshoot, and relaxation time were measured by rapidly pulling (14 mm/s) the sensor to 50% strain, holding for 10 seconds, and releasing at the same rate back to 0% strain and held for 20 seconds before starting the next cycle. The response time was determined by thresholding to once the sensor response passed 3 standard deviations of the baseline resistance.[2] We quantified the overshoot upon reaching 50% strain as a percentage of the overall signal change and determined the relaxation time based off a 10% tolerance of the baseline value once the sensor has returned to 0% strain. The sensor undergoes 10 cycles in total, with the average values across all 10 cycles being reported. The sensor remained untouched for at least 5 minutes post-conditioning to allow the elastomer to rebound back to a stable baseline. If

necessary, the sensor was readjusted to be taut upon starting the tensile pulling. A 10 minute rest was allowed between strain sensitivity and response latency testing.

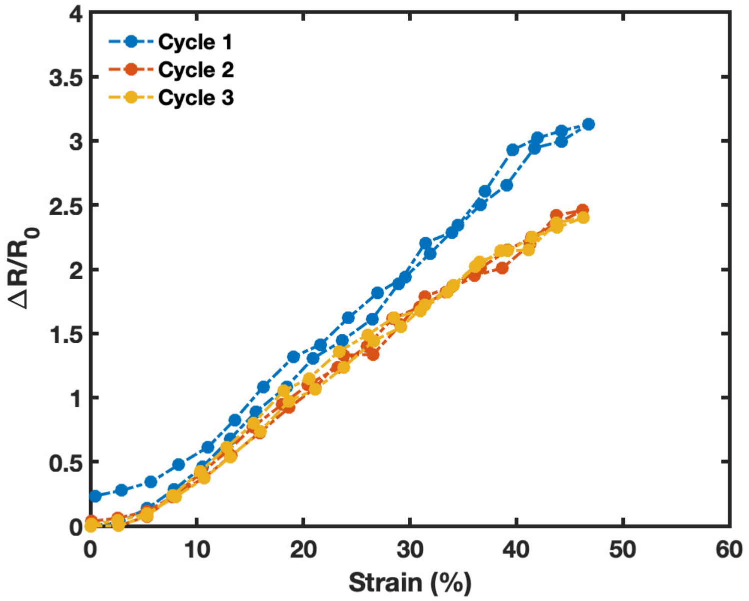

Figure S2. Full sensitivity data for a representative sensor collected as described under electromechanical characterization.

Stretchability of the sensors were measured using the same tensile testing apparatus where each sensor as incrementally strained at 10s intervals until the strain resulted in loss of electrical connection. The average normalized change in resistance of the interval is shown in the data.

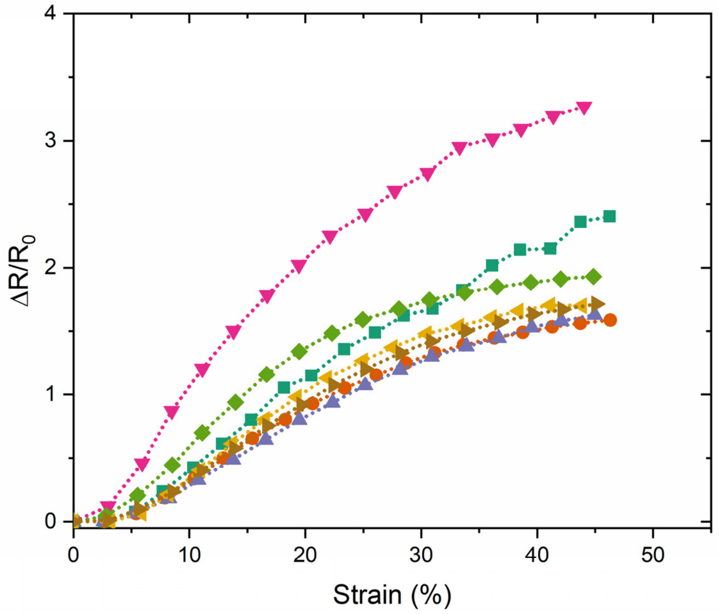

Figure S3. Sensitivity curves for all unencapsulated sensors (N=6).

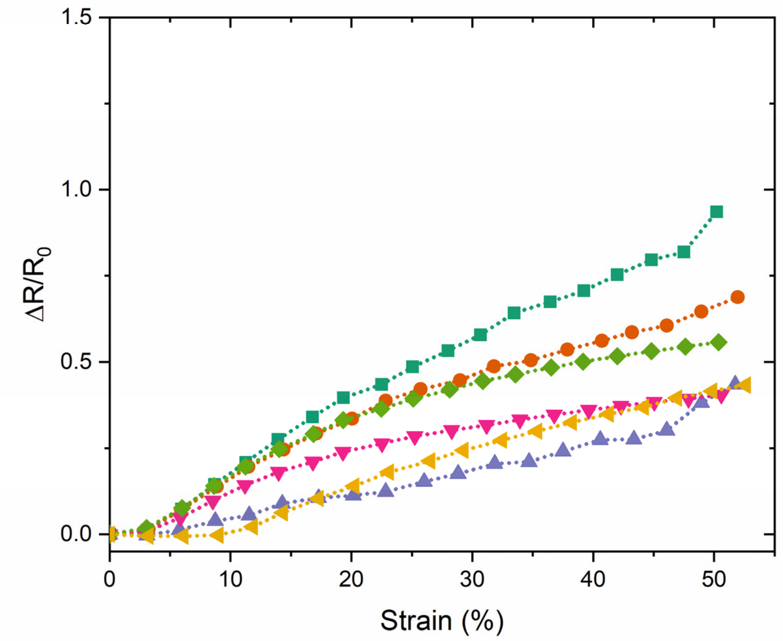

Figure S4. Sensitivity curves for all encapsulated sensors (N=6).

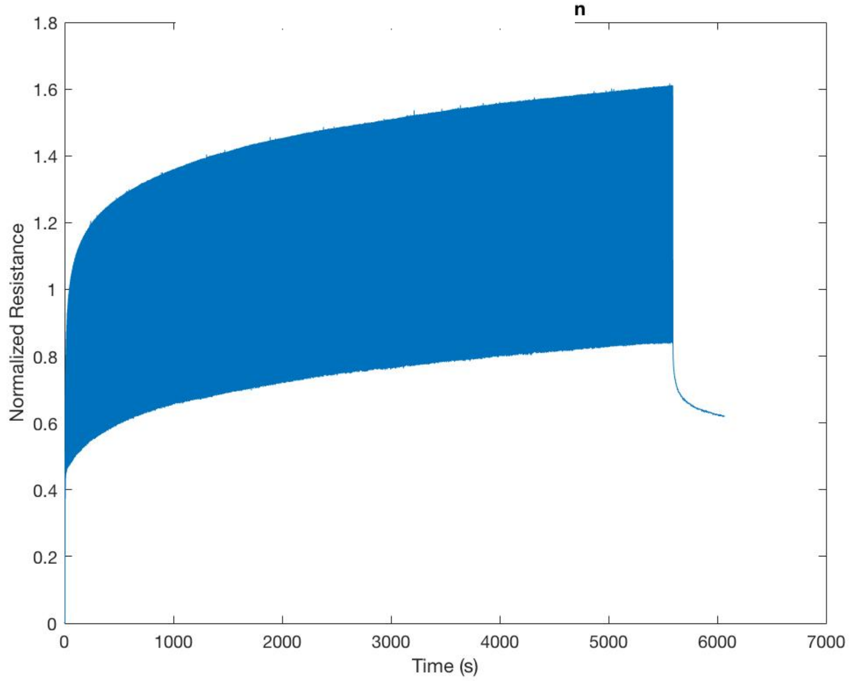

Figure S5. Full cycling data for unencapsulated sensor with without preconditioning.

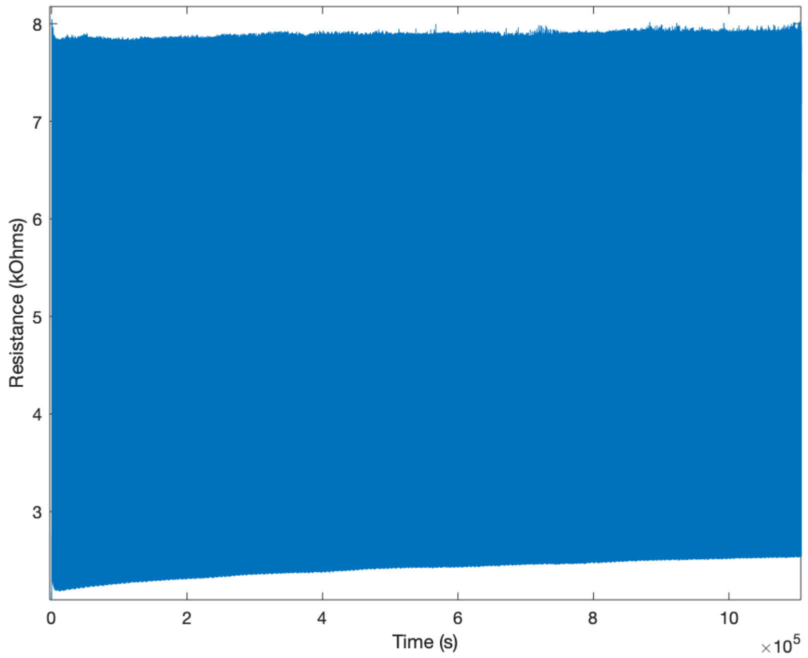

Figure S6. Full cycling data for unencapsulated sensor (pre-fracture) with 50 cycles at 100% strain preconditioning done prior (unshown).

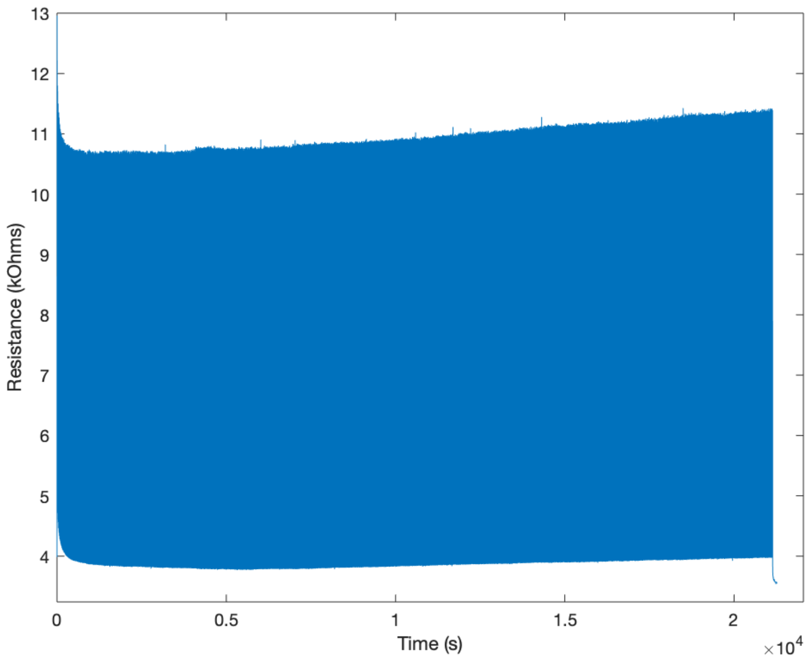

Figure S7. Full cycling data for encapsulated sensor (pre-fracture) with 50 cycles at 100% strain preconditioning done prior (unshown).

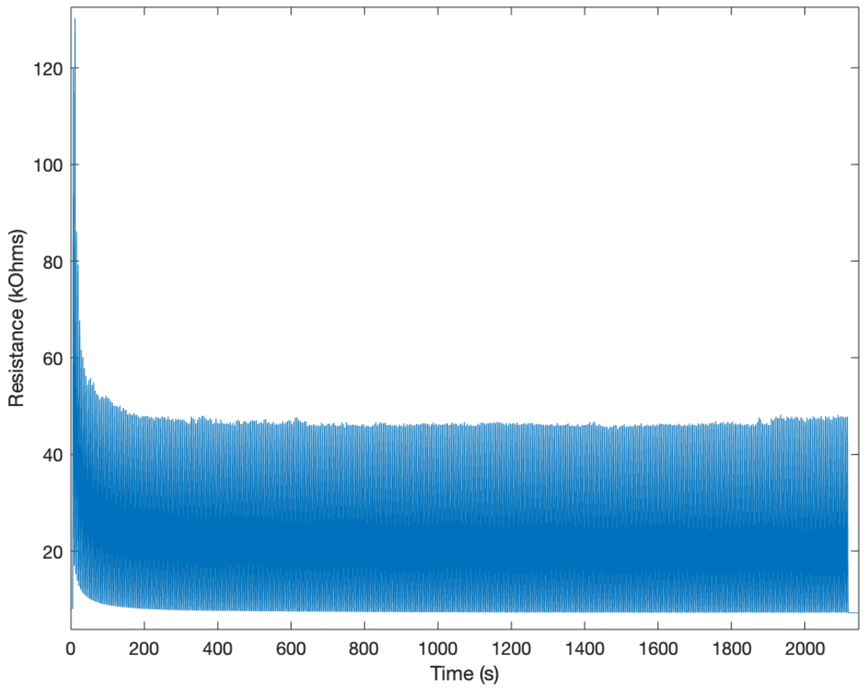

Figure S8. Full post-fracture conditioning data for an unencapsulated sensor.

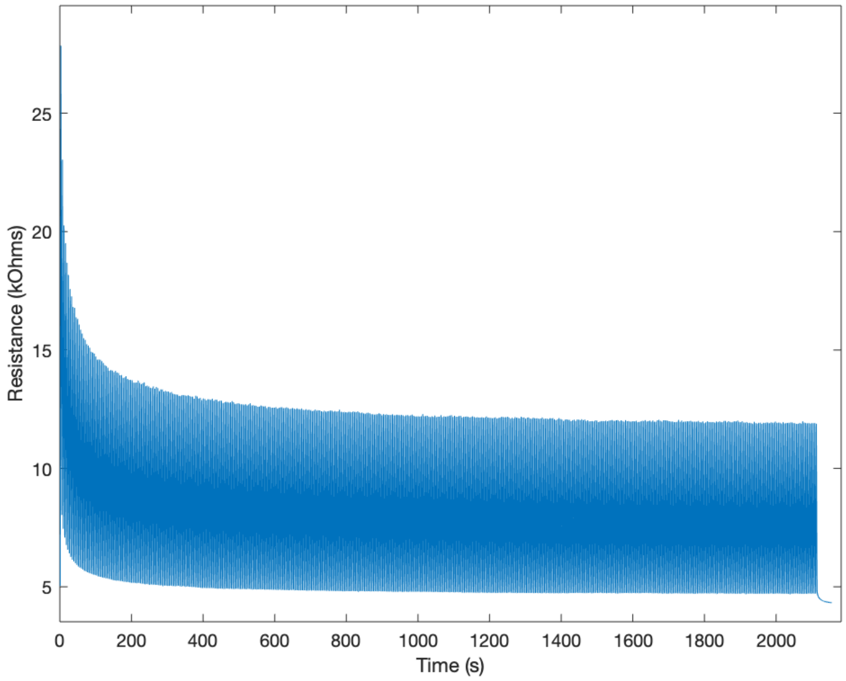

Figure S9. Full post-fracture conditioning data for an encapsulated sensor.

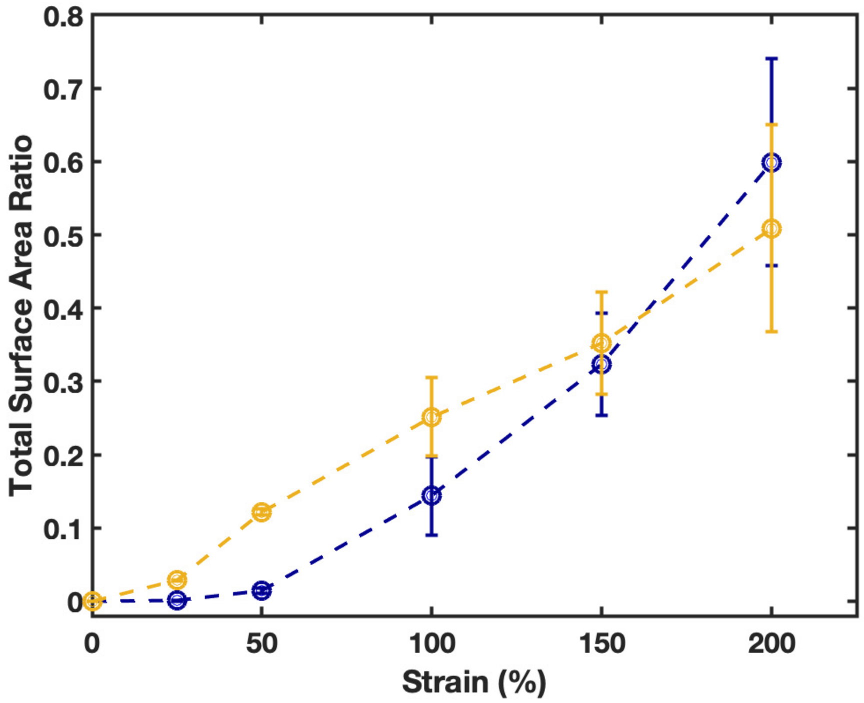

Figure S10. The total surface area ratio of metal to cracks within the field of view is the same for both unencapsulated and encapsulated samples. Differences are shown to be statistically insignificant across each strain position.

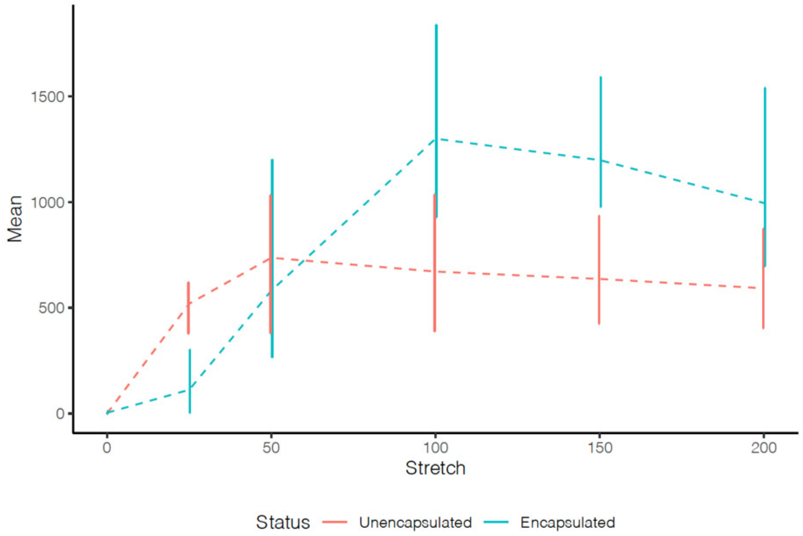

Figure S11. Plot of the mean number of cracks with increasing strain where the bars represent the spread from minimum to maximum values at the respective strain point, not standard deviation.

Consult supplemental crack analysis files for additional details on multivariate statistical analysis.

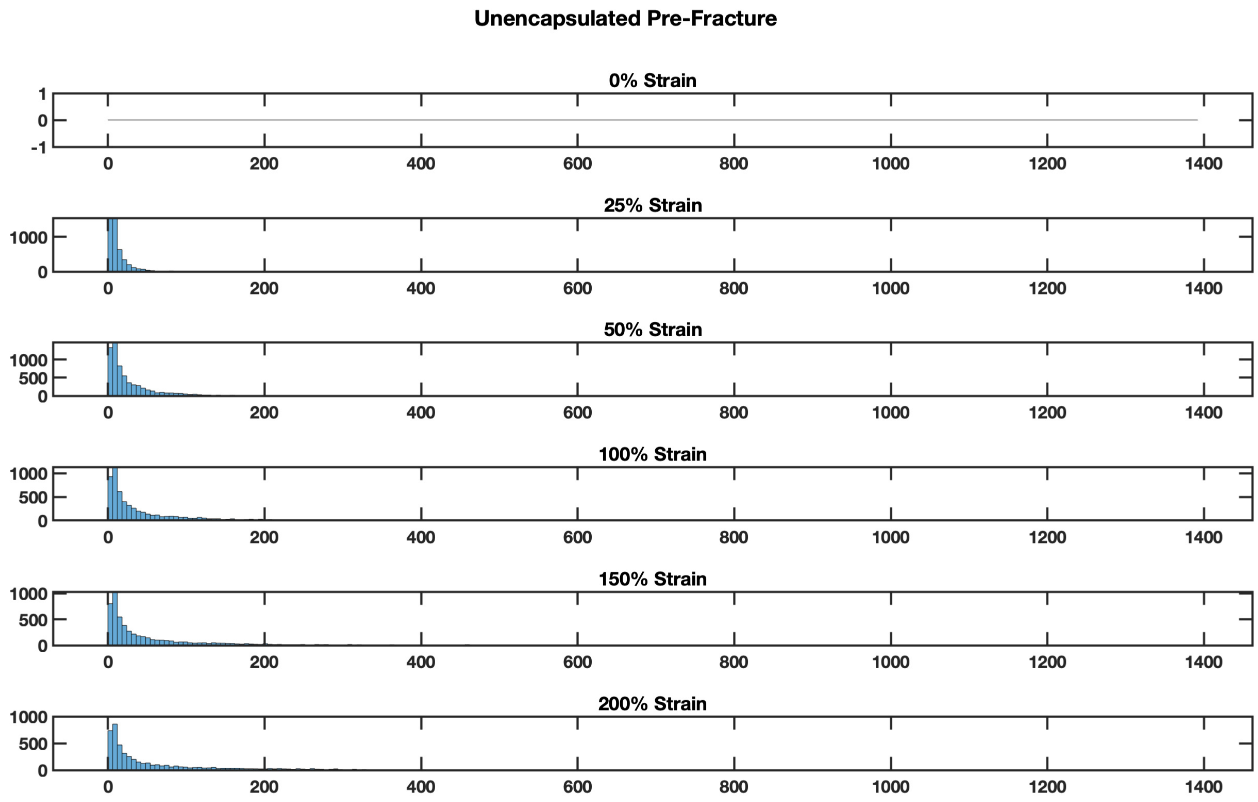

Figure S12. Distribution of crack size (crack area μm²) for the pre-fractured unencapsulated film across strain points.

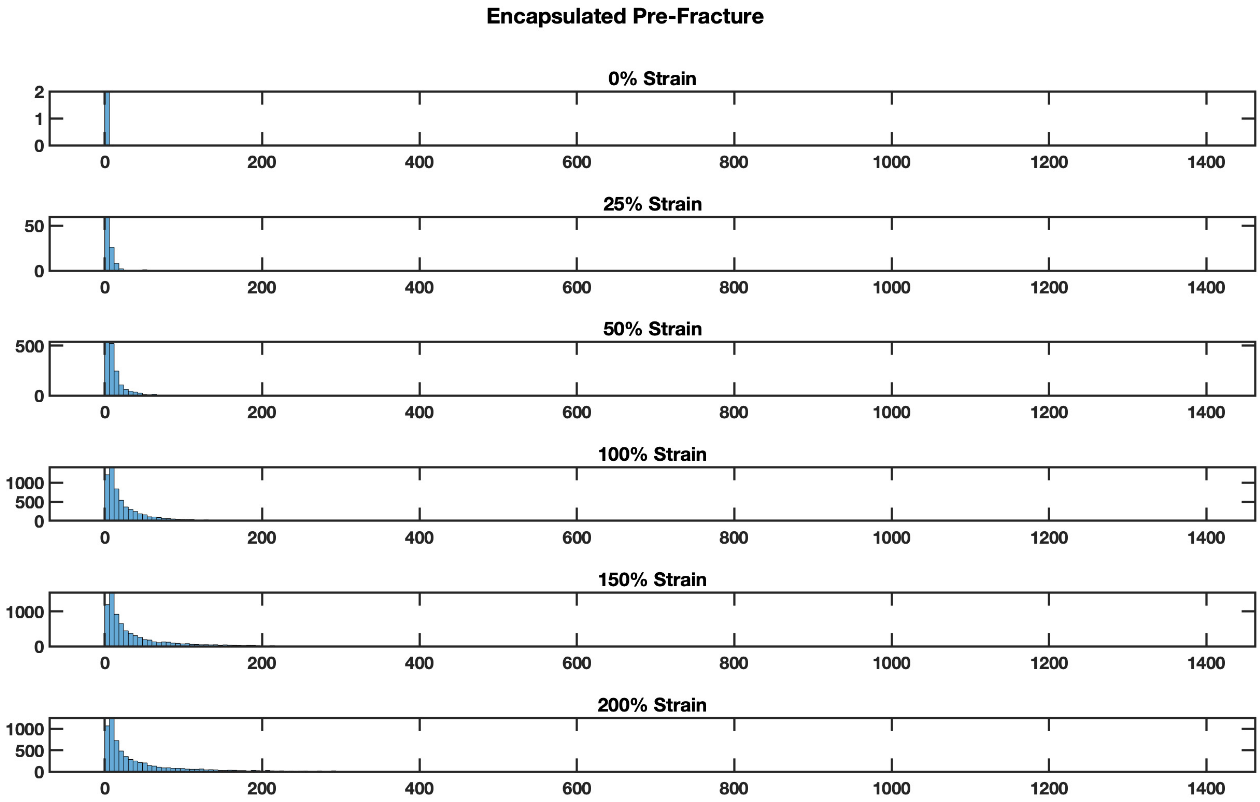

Figure S13. Distribution of crack size (crack area μm²) for the pre-fractured encapsulated film across strain points.

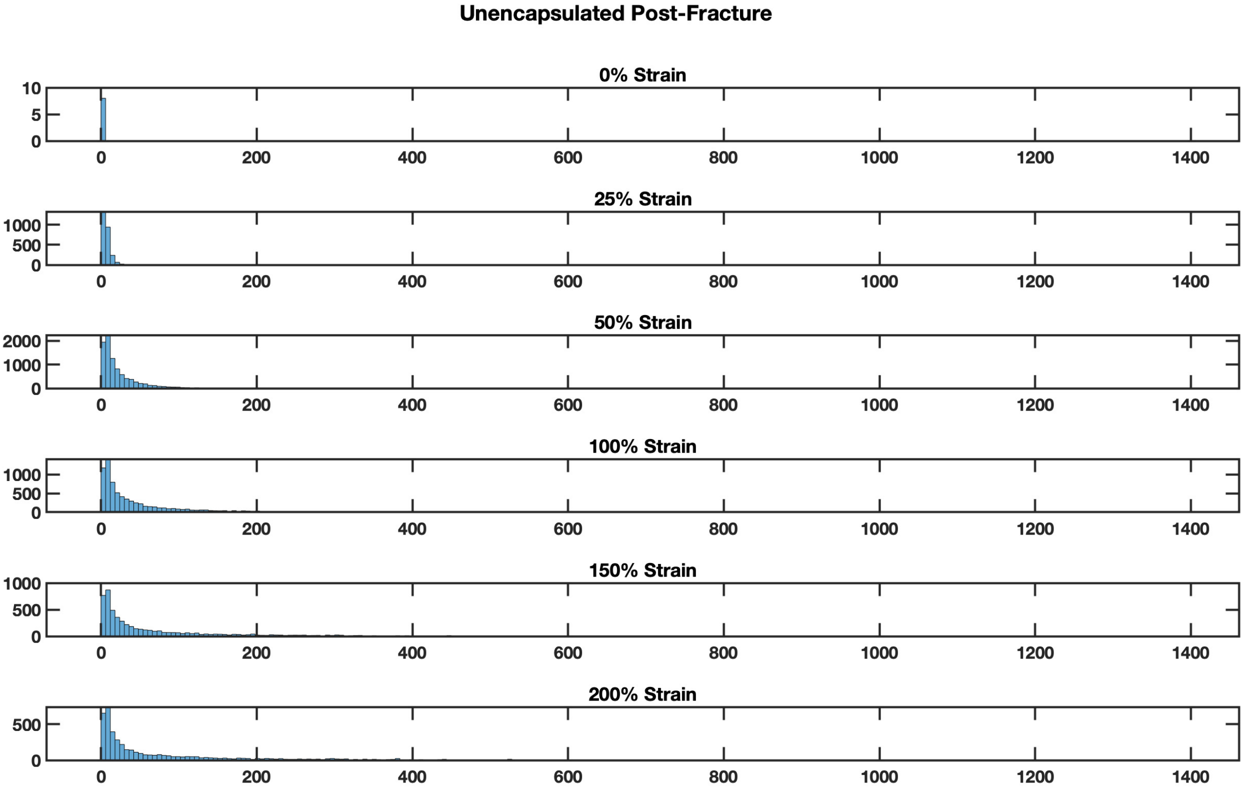

Figure S14. Distribution of crack size (crack area μm²) for the post-fractured unencapsulated film across strain points.

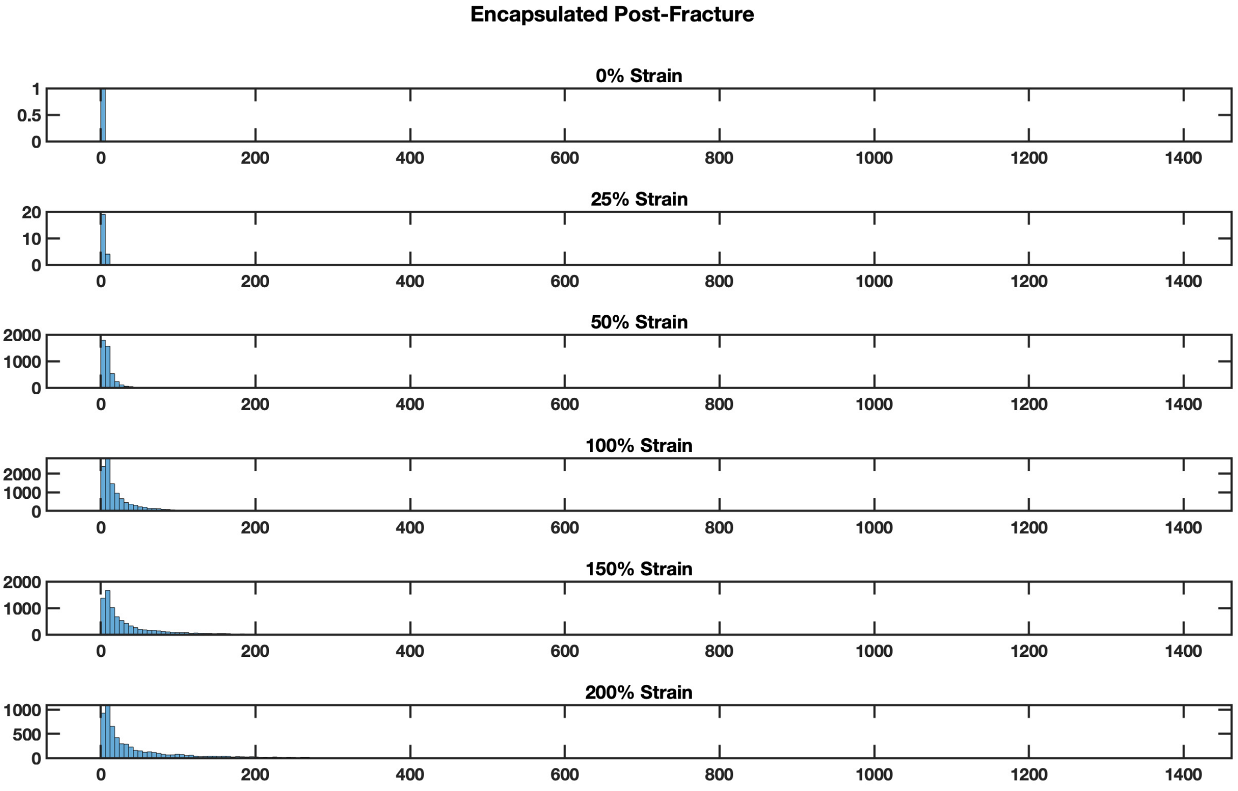

Figure S15. Distribution of crack size (crack area μm²) for the post-fractured encapsulated film across strain points.

References

1. Kang, D.; Pikhitsa, P.V.; Choi, Y.W.; Lee, C.; Shin, S.S.; Piao, L.; Park, B.; Suh, K.-Y.; Kim, T.-I.; Choi, M. Ultrasensitive mechan-

ical crack-based sensor inspired by the spider sensory system. *Nat. Cell Biol.* **2014**, *516*, 222–226, doi:10.1038/nature14002.
2. Atalay, A.; Sanchez, V.; Atalay, O.; Vogt, D.M.; Haufe, F.; Wood, R.J.; Gafford, J.B. Batch Fabrication of Customizable Silicone-Textile Composite Capacitive Strain Sensors for Human Motion Tracking. *Adv. Mater. Technol.* **2017**, *2*, 1700136, doi:10.1002/admt.201700136.
